# Effectiveness of Communication Skills Training in Medical Students Using Simulated Patients or Volunteer Outpatients

**DOI:** 10.7759/cureus.26717

**Published:** 2022-07-10

**Authors:** Adlene I Adnan

**Affiliations:** 1 Internal Medicine: Dermatology, Harrogate District Hospital, Leeds, GBR

**Keywords:** medical students, learning theories, volunteer outpatients, stimulated patients, medical education, communication skills

## Abstract

Communication skills are the vital basis for patient-doctor interactions in undergraduate medical education. With excellent patient-physician interaction and communication, patients will likely build better rapport and trust with the physician. This allows all the necessary information to be divulged with the reassurance of confidentiality and devise appropriate investigations and treatment plans that patients would be more inclined to follow. The most common and effective ways of teaching communication skills to medical students are by using simulated patients and volunteer outpatients. However, which types of patients to use for better development of practical communication skills training. Establishing the demonstrable difference between using two kinds of patients would refine the training scheme for students. This would produce doctors that have practical communication skills and enhance their care to assist patients on their road to recovery or palliative care. This review compares and establishes the effectiveness of medical students’ communication skills training using simulated patients and volunteer outpatients about the adult learning theories.

This research is carried out following a critical review of internationally reputed guidelines from the World Health Organisation (WHO) and the General Medical Council (GMC). Several search terms were used on various online databases such as Medline (Ovid), PubMed, and Academic Medicine. A thorough selection process was applied using the inclusion and exclusion criteria to narrow the search. Four studies related to this review’s aim were collected and critically analyzed. The methods of obtaining the studies were structured using the PRISMA guidelines. The studies showed that one study favored volunteer outpatients while the other preferred having simulated patients. Another study showed that students considered both types of patients essential for communication skills training.

All the studies presented the strengths and weaknesses of both simulated and volunteer outpatients. Discussion of the validity of all analyses was based on the CASP criteria. Study design, sample selection, and biases were scrutinized for each study. Various adult learning theories were used to correlate the effects of the communication skills training. In conclusion, simulated patients are more useful for pre-clinical years, intimate examination, and giving instructions about the physical examination. Whereas volunteer outpatients are put to better use in clinical years to incorporate more medical aspects such as obtaining a differential diagnosis, management of illness, and procedural techniques. Introducing different types of patients based on their study progression and topic of discussion could be adapted.

## Introduction and background

Communication skills encompass the ability to impart information by speaking, writing, or using any other medium as efficiently as possible [1]. These communication skills are essential for patient-doctor interactions in undergraduate medical education. With excellent patient-physician interaction and communication, patients will likely build better rapport and trust with the physician. This allows them to divulge all the necessary and sensitive information with the reassurance of patient-doctor confidentiality, allowing appropriate investigations and a treatment plan to be devised. Patients are also likely to adhere better to the given treatment plans and risk factor control and achieve better health outcomes [2,3]. Thus, patient interaction would help students learn more about communication and clinical reasoning skills with the help of constructive feedback [2]. 

This is highly relevant as the Accreditation Council for Graduate Medical Education has established the importance of communication and interpersonal skills as the foundation competency requirement for residents and practicing physicians [3]. Therefore, it is imperative that these communication skills techniques are to be taught in the best way possible to promote learning. However, the best course of conveying these essential skills has remained uncertain.

In medical schools, the most common and effective ways of teaching communication skills to students are by using simulated patients (SPs) and volunteer outpatient (VOs). SPs are standardized patients trained to act as actual patients to simulate clinical problems; meanwhile, VOs are actual patients with clinical symptoms who voluntarily participate in learning opportunities when invited [2-5]. This allows the medical students to elicit comprehensive histories, practice physical examination skills, give patient instructions like inhaler techniques and break bad news with a more patient-centered approach [2-5].

For decades, SPs have been more than often used to teach medical students communication skills. This has been proven to bring about positive outcomes based on the satisfaction rate given by medical students and their examination scores [3]. This is because SPs provide standardization of assessment to focus on the accuracy and consistency of students’ performance when given different examiners [5]. Furthermore, simulation-based medical education would allow students to increase their confidence and enhance their techniques before facing the real-world scenario. This includes surgical procedures, advanced care life support training, intimate examinations, and consultation skills [2-5]. This would reduce the potential harm to actual patients as students would practice and develop the necessary skills beforehand in a safe learning environment with valuable feedback from SPs and peers [4,5].

However, SPs would also present their limitations. The element of authenticity in SPs are always questionable as they would often appear artificial when it comes to expressing their emotions. Hence, students may feign an empathic demeanor and responses in their encounters with SPs to impress the faculty examiner rather than be genuine [4,5].

In contrast, VOs present genuine emotions and concerns drawn from their past and current experiences with medical patients, which give interactions much more authenticity and help bridge the gap between simulated scenarios and real-world patients. VOs would also present an actual complaint and illness that would assist and reinforce students’ medical knowledge [2,3]. However, some students complained that having actual patients would emphasize the medical aspects more, which would gradually prevent medical students from being empathetic toward patients’ distress to have an interrogative approach to patients [2,4].

Hence, establishing the demonstrable difference between using both types of patients would refine the training scheme for students. Ultimately, this would produce doctors with practical communication skills and enhance their ability to care for patients on their road to recovery or palliative care.

Aim

To compare and establish the effectiveness of communication skills training in medical students using simulated patients or volunteer outpatients regarding the adult learning theories.

## Review

Method

We researched via textbooks, journals, and internet searches to gain more background knowledge on this topic of interest. We studied internationally reputed guidelines from WHO and GMC beforehand as well. To establish the feasibility and importance of this review, a literature search was done using the medical databases with a minimum target of 4 studies. Medline (Ovid), PubMed, and Academic Medicine were the medical databases that showed relevant search results. Specific search terms were used in those medical databases (Tables 1-3).

**Table 1:** Search terms used in Medline (Ovid)

Keywords	Number of Results
(Communication)	76119
(Communication) AND (Medical students)	905
(Communication) AND (Medical students) AND (Patient simulation)	603
(Communication) AND (Medical students) AND (Patient simulation) AND (Volunteer outpatients)	108

**Table 2:** Search terms used in PubMed

Keywords	Number of Results
(Communication skills training)	17193
(Communication skills training) AND (Medical student)	2727
(Communication skills training) AND (Medical student) AND (Real patients)	101
(Communication skills training) AND (Medical student) AND (Real patients) AND (Simulated patients)	43

**Table 3:** Search terms used in Academic Medicine

Keywords	Number of Results
(Simulated patients)	160
(Simulated patients) AND (Medical students)	100
(Simulated patients) AND (Medical students) AND (Real patients)	40
(Simulated patients) AND (Medical students) AND (Real patients) AND (Communication skills)	13

**Figure 1:**
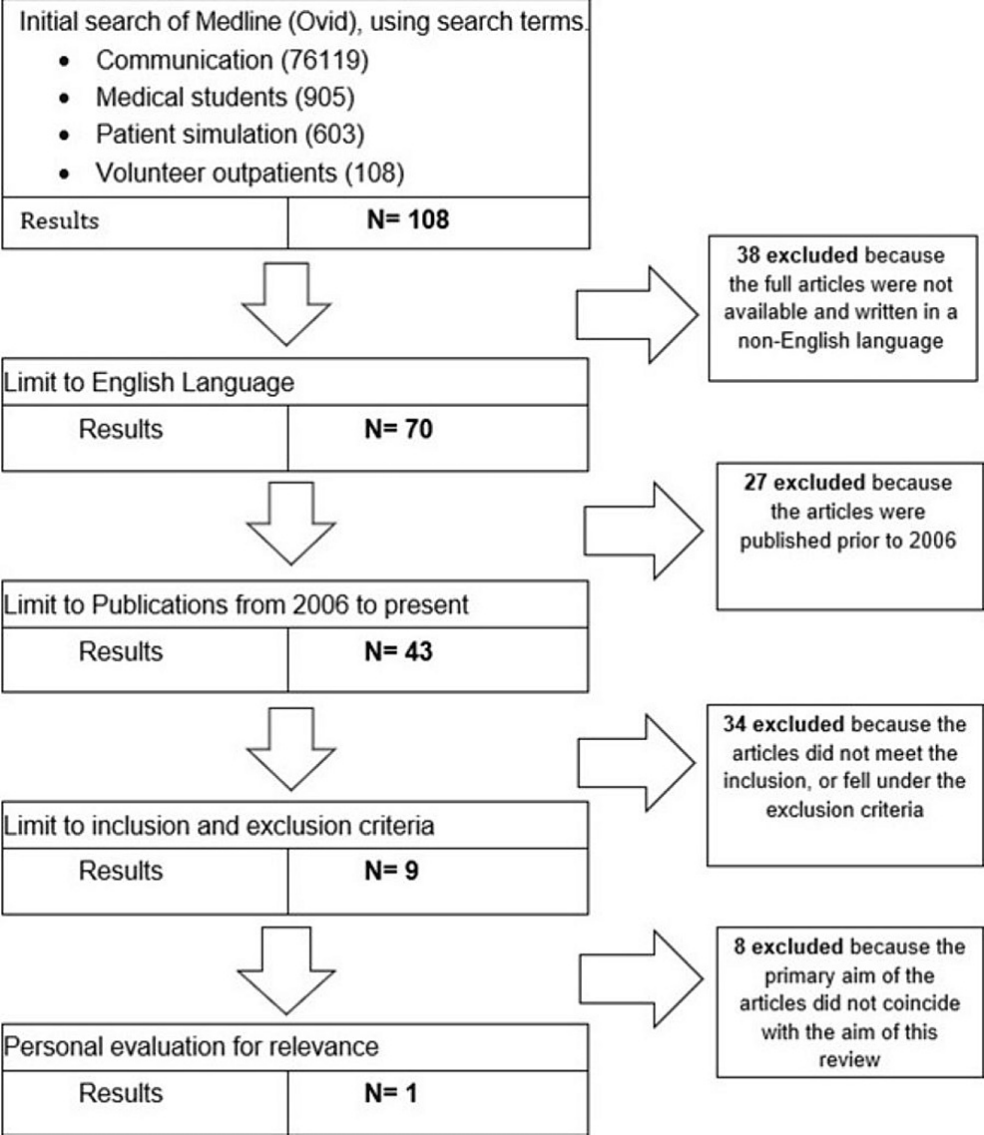
PRISMA flow diagram for Medline (Ovid) search

**Figure 2:**
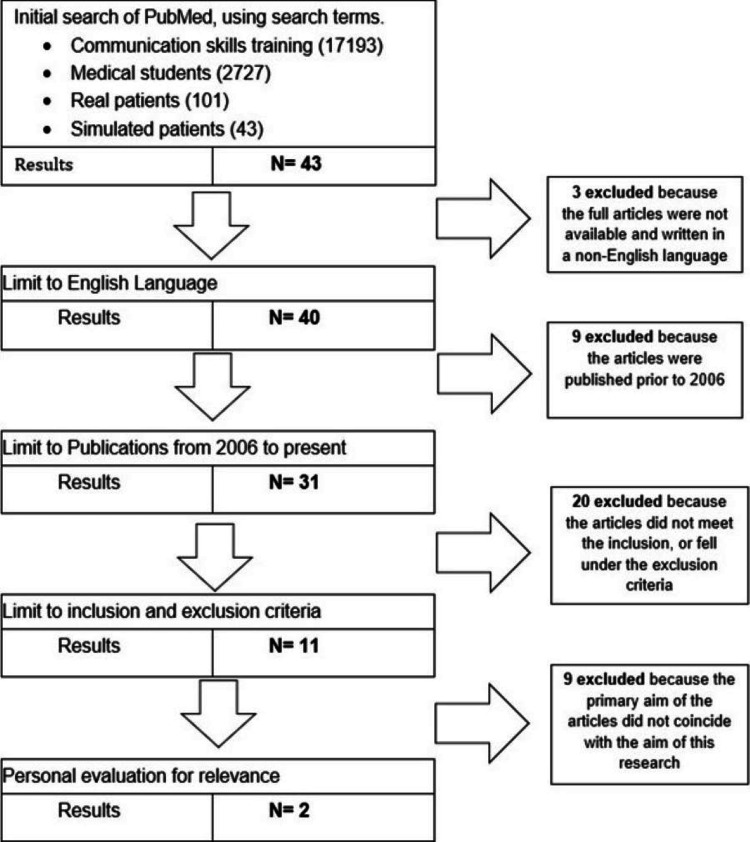
PRISMA flow diagram for PubMed search

**Figure 3:**
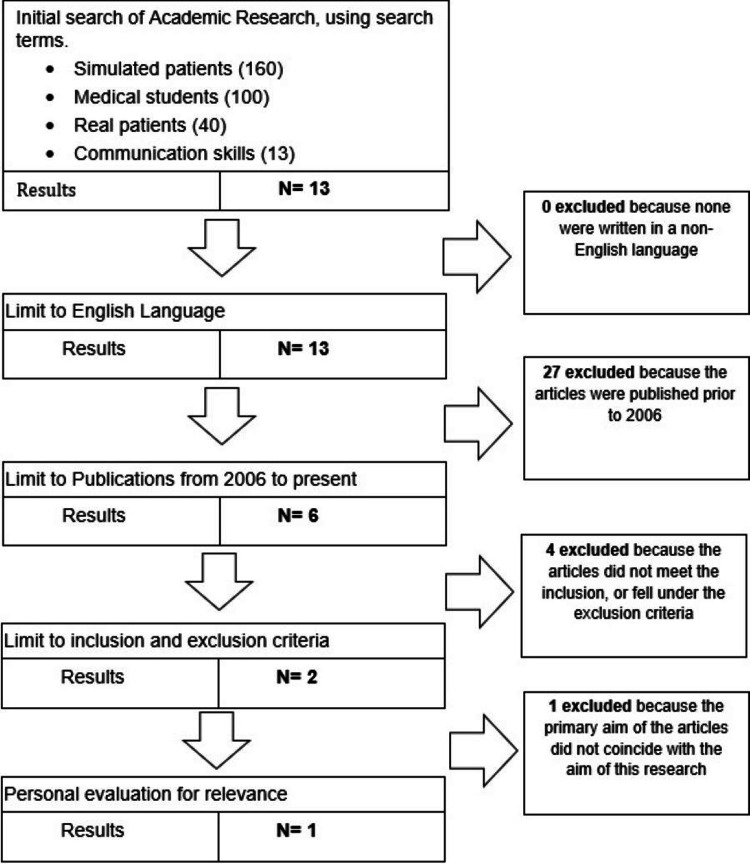
PRISMA flow diagram for Academic Medicine search

The articles obtained were narrowed down through a selection process. The titles and abstracts were thoroughly evaluated for their relevance to the aim of this literature review using inclusion and exclusion criteria (see table 4). Structured PRISMA Guidelines were used to clearly demonstrate the methods of obtaining the chosen studies [6] (see figures 1-3).

**Table 4:** Inclusion and Exclusion Criteria for studies

Criteria	Inclusion	Exclusion
Recruitment criteria	Medical students from Year 1 to 6	Qualified doctors, nurses, and other medical healthcare members
Language	English	Non-English
Publication Dates	Published dates of the past 10 years till present	Published dates of more than the past 10 years
Topics at Evaluation	History taking, Physical examinations, Procedural Techniques, Counselling, and clinical management skills.	Writing Skills
Others:	Studies are free to access using University of Liverpool library resources and Google.	Studies without fully available articles.

Results

One study was obtained from Medline (Ovid), two studies were selected from PubMed and one study was taken from Academic Medicine [2-5]. The list of studies chosen is shown in Table 5 seen below. Table 6 shows the summary of the four chosen articles including the author, title of the paper, year, aim, and key findings. All papers matched the CASP criteria that were used as guidance to check the validity, relevance, and results of the studies chosen [7].

**Table 5:** List of studies chosen for critical appraisal

Author / Year	Search Engine	Main Findings
Clever SL et al.(2011) [3]	Medline (Ovid)	Interactions with simulated patients are less effective than with volunteer outpatients in communication skills training.
Jabeen D (2013) [5]	PubMed	Interactions with simulated patients are more effective than with volunteer outpatients in communication skills training.
Elley CR et al. (2012) [4]	PubMed	Interactions with simulated patients are more effective than with volunteer outpatients in communication skills training.
Bokken LM et al. (2009) [2]	Academic Medicine	Interactions with simulated patients and volunteer outpatients are equally effective in communication skills training.

**Table 6:** Summary of the four articles, including author, the paper's title, year, aim, and key findings.

Author	Title of paper	Year	Aim	Key Findings	
				Results on the use of volunteer outpatients	Results on the use of simulated patients
Clever SL et al. [3]	Medical Student and Faculty Perceptions of Volunteer Outpatients Versus Simulated Patients in Communication Skills Training	2011	To determine whether medical students and faculty perceive differences in the effectiveness of interactions with volunteer outpatients versus simulated patients in communication skills training.	Students find better interaction with volunteer outpatients in terms of friendliness, comfort in the interview, amount of learning, opportunity to build rapport, and overall meeting of communication skills training needs. Female students gave higher mean ratings than male students for the opportunity to build rapport and for the interviewer’s meeting their educational needs.	Students felt that the simulated patients deliberately withheld information necessary and found it hard to cooperate with them, unlike volunteer outpatients where information was readily given.
Jabeen D [5]	Use of Simulated Patients for Assessment of Communication Skills in Undergraduate Medical Education in Obstetrics and Gynaecology	2013	To compare the effectiveness of simulated patients with real patients through undergraduate students' results of Mini-CEX encounters and their opinions.	Volunteer outpatients were less in favor because this study was focused on sensitive topics and there were risks of students’ performance being distressing to them. They also had the tendency to be less available in certain situations and some were not willing to participate in examinations where they are exposed to many students.	Most of the students preferred using simulated patients for their communication skills assessment. Simulated patients were readily available for examinations. They gave students opportunities to practice their communication skills in a low-risk environment. The competency, accuracy, and consistency of students’ performance can be established better as the other variables like simulated patients and examiners were controlled.
Elley CR et al. [4]	Effectiveness in Simulated Clinical Teaching in General Practice	2012	To assess the effectiveness of ‘simulated’ general practice clinics using actors, compared with standard community-based general practice attachments in medical undergraduate education.	Students felt more confident in dealing with upper respiratory tract infections, screening in general practice, administering injections, and managing illness in patients’ houses compared to those that were placed in the simulated clinics. Therefore, even though simulated patients may assist in the development of communication skills, they may also be used to supplement volunteer outpatients to improve in managing common conditions and procedures.	Interactions with simulated patients in simulated clinics improved students’ confidence in history taking, communication skills, and the ability to recognize depression significantly more than those who had interactions with volunteer outpatients.
Bokken LM et al. [2]	Students’ Views on the Use of Real Patients and Simulated Patients in Undergraduate Medical Education	2009	To determine students’ views about the strengths and weaknesses of real patient interactions as opposed to simulated patient interactions in the undergraduate medical curriculum.	Preparation was made better for volunteer outpatients encounters than simulated patients because students felt more responsible towards them and it gave a greater emphasis on learning medical knowledge rather than communication skills. Hence, they felt as if they were less empathetic and in turn became robotic doctors that are keen on getting the diagnosis, rather than comforting the patients’ worries. Students also suggested integrating more volunteer outpatients in learning physical examinations to distinguish between normal and abnormal physical findings.	Simulated patients were useful in preparation for real patient interactions. Some suggested a gradual increase of simulated patient encounters in the first two years of medical school. Simulated patients were very beneficial in learning intimate examinations, introducing physical examination in the consultation, and practicing verbalizing instructions to patients with regards to the examination. Most students found feedback given by simulated patients to be more useful than feedback given by volunteer outpatients because simulated patients are well-trained in detecting even the smallest improvements while volunteer outpatients would often give little feedback.

Discussion

Validity of Study

Issues addressed: Even though all studies thoroughly evaluate similar issues, studies by Clever et al. [3] and Elley et al. [4] differ slightly from studies by Jabeen [5] and Bokken et al. [2] in terms of the medical topic that the communication skills were tested. Since the subjects in Clever et al. [3] study were first-year medical students and the subjects in Elley et al. [4] study were in their first clinical year, the range of medical discussion was based on general medicine topics. This contrasted with the other studies, such as the Jabeen [5] study, where fourth-year MBBS students were assessed on communication skills solely in Obstetrics and Gynaecology, and the Bokken et al. [2] study, where fourth and fifth-year medical students were scheduled in for their clinical specialties rotations.

In terms of the titles of the studies, the studies by Clever et al. [3] and Bokken et al. [2] stated their title precisely about the aim to evaluate the communication skills training using SPs and VOs. However, the studies by Jabeen [5] and Elley et al. [4] did not mention their intention to compare both types of patients as their titles referred to the effectiveness of using only SPs. However, their choices are only evident in their content as comparisons were made. Therefore, both studies should improve on their title to prevent misunderstandings on the study by the author as it might not fit in this review.

The method of evaluating medical students’ effectiveness and performance with the SPs and VOs differs in all four studies. The studies by Clever et al. [3] and Jabeen [5] had the assistance of preceptors, which were the faculty members that observed the communication skills training session. In both these studies, quantitative data was obtained from preceptors, aside from the students. The study by Jabeen [5] used the preceptors to evaluate the students’ performance on an assessment sheet called the Mini-CEX. It was calculated to produce the quantitative data for the study without the students’ input.

Meanwhile, the study by Clever et al. [3] collected data from both the students’ and preceptors’ points of view as well. It provided it in a separate statistical measurement that would not use the preceptors’ data in this literature review as it does not coincide with the aim of this review. However, the studies by Elley et al. [4] and Bokken et al. [2] collected information based on students’ perceptions, and there were no preceptors in these studies.

Study Design: Out of all the other studies, Bokken et al. [2] used a retrospective cohort, while the other used prospective cohort studies. Even though a retrospective cohort study is inexpensive and less time-consuming, certain factors such as external exposure and factors cannot be controlled. Therefore, the outcome was difficult to assess, which may compromise the reliability of the study [8]. In this study, the participants had prior exposure to simulated patients in their pre-clinical years and now have exposure to real patients. This could have biased their perceptions as it might be difficult for students to distinguish to what extent interactions with SPs have influenced their encounters with actual patients. Confounding bias could be appreciated here. Therefore, it would have been ideal if the evaluation of simulated patients’ and real patients’ strengths and weaknesses were done on students who were not previously biased, for example, first-year students in this case [2].

Various methods to assess the effectiveness of medical students’ communication skills using the two different patient types were evaluated. Studies by Clever et al. [3] and Jabeen [5] used both quantitative and qualitative methods to obtain data. In contrast, studies by Elley et al. [4] and Bokken et al. [2] used quantitative and qualitative methods, respectively. Clever et al. [3] study developed questionnaires with the guidance of the Maastricht Assessment of Simulated Patients. Additional items regarding the learning environment were incorporated into the questionnaire to ensure accuracy. This shows more work has been done to evaluate the standards and reliability of the questionnaire.

On the contrary, a Mini-CEX questionnaire was used in Jabeen [5] study using a rating scale developed by the American Board of Internal Medicine in 1990. Since it was created a long while ago, the reliability and accuracy of the questionnaire cannot be fully determined as new medical treatments could be missed in the history-taking section [3]. Similarly, the questionnaire utilized in Elley et al. [4] study had been used for several years in a general practice attachment. Unfortunately, the validity of the questionnaire has not been checked for the research because it would have caused an additional burden to this study [4]. This might have compromised the accuracy of the data collected. In the study by Bokken et al. [2], an interview was conducted to obtain students’ perceptions about the topic. It was recorded with a digital audiotaping system for later transcription. The interviews were conducted in Dutch and were translated to English. The transcripts were sent to the students in the focus group for amendments and final approval [2]. This is an excellent way of preventing any information bias in case ideas come across differently from what was meant. Since it was translated from a different language, there was a higher chance of discrepancy if the students did not re-evaluate what they had said.

Sample selection and biases: The sampling method of studies by Clever et al. [3] and Elley et al. [4] aimed to achieve demographic variation for a good representation of the population. Students were also stratified based on gender to allow almost equal differences between male and female students. Clever et al. [3] study filtered their recruited VOs to ensure they could speak English and were not perceived to have any personality disorder. However, it failed to obtain different demographic participants because most VOs were from a retirement-aged population. These individuals were likely to be available during the daytime when these activities were being done. Additionally, the recruitment techniques involved could have resulted in more amiable outpatients than the simulated patients, as indicated by the students’ higher “friendliness” ratings [3]. Hence, this study had a high sampling bias.

In terms of sample size, Clever et al. [3] study had a relatively large sample size. It had the largest sample size of 121 students compared to the other studies, with a low attrition rate of 2%. This makes the findings significant to reflect the population, especially those in medical school. Meanwhile, the study by Jabeen [5] had a smaller sample size of 94 students with a 0% attrition rate. Even though there were no dropouts, the small sample size would question the reliability of this study. Elley et al. [4] had 106 students follow through to complete the study with an 11% attrition rate. Based on outcomes of previous years, 126 students would allow a sufficient number to detect a 10-20% difference between the groups. Hence, there is a significant attrition rate due to its small sample size [4]. Out of all the students invited to participate in the Bokken et al. [2] study, only 48% agreed to join, indicating a small sample size of 38 students with no dropouts. Therefore, the studies by Bokken et al. [2], Jabeen [4], and Elley et al. [4] may cause results to be insignificant.

In all the studies, students were not blinded to the patient types [9]. This is because there were no relevant educational or testing settings that were to come about if students were not aware of the types of patients they were interviewing [2-5]. Secondly, the research focused on students’ perceptions of patient types, so revealing this information was necessary. Although that was all true, this could have led to favorable biases by the students towards certain types of patients based on their experiences [3]. However, in Jabeen’s [5] study, even though the students were aware of the patient types, the preceptor conducting the assessments on the students with the VOs was unaware of the score the other preceptors would rate when students were having their encounters with the SPs. Thus, there was an element of blinding here to prevent observer bias. Meanwhile, Elley et al. [4] used a researcher who was not involved in the outcome of the studies and carried out a computer-generated block assessment to quantify the data. This would have reduced any ascertainment bias in the study.

Furthermore, Bokken et al. [2] study was an interview conducted on students’ experience with both types of patients. This would create a recall bias whereby the students might not be sure about how practical their past training was with simulated patients. Therefore they preferred their current interactions with actual patients. Other than that, the questions that were being asked in the interview suggested interviewer bias. For example, the researchers asked about the strengths and weaknesses of simulated patients and not the way around for VOs. This would subconsciously influence the subject into giving answers skewed towards the interviewer’s own bias, hence causing response bias.

Results of Study

Measurement of effectiveness: The measurement of ‘effectiveness’ is very vague and challenging to analyze. Different types of questionnaires would have revealed different sizes of ‘effectiveness.’ Jabeen’s [5] study focused more on students’ performance as a measurement of ‘effectiveness.’ On the other hand, other studies based it on students’ perceptions and experiences [2-4]. This variation is because many people have their respective ways of learning, hence leading to several adult learning theories present to this day.

**Figure 4:**
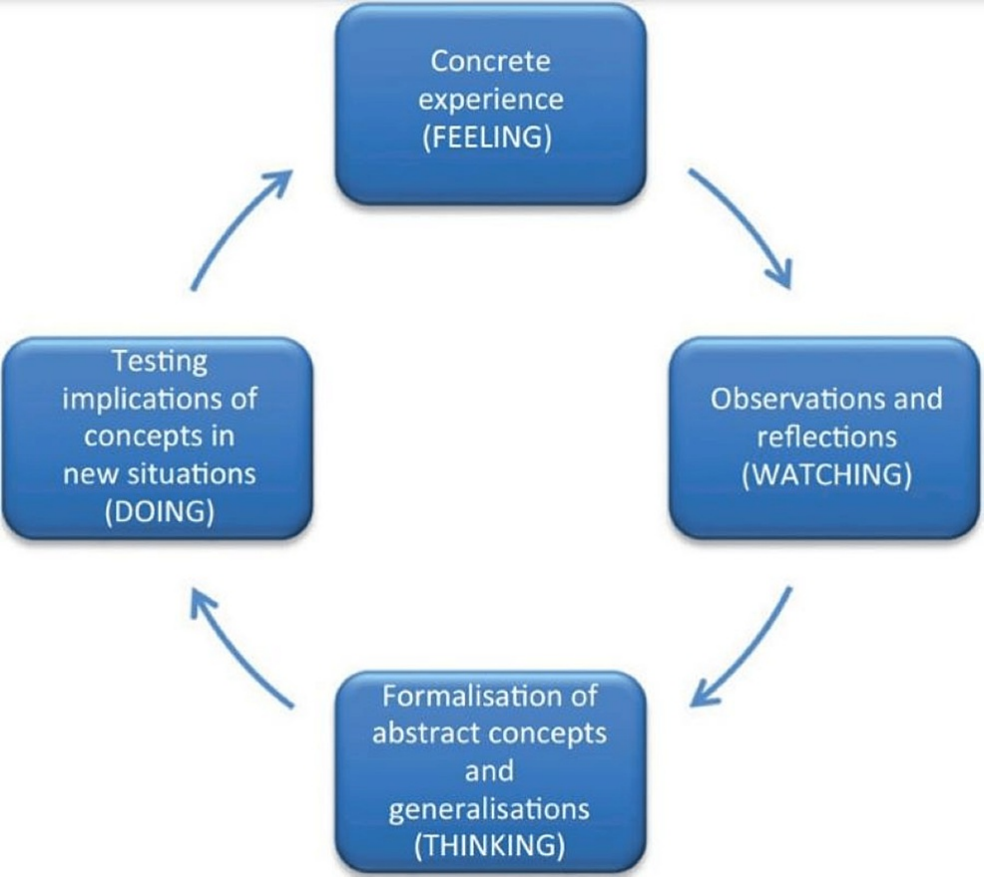
The Kolb Cycle 1984 Ref no- [10] Permission to use image granted.

Clever et al. [3] study used The Kolb Cycle 1984 (Figure 4) [10] to evaluate how effective the learning process was. Students were allowed to interview both types of patients and rate their interaction based on comfort level, friendliness, amount of learning, relationship building, and overall interaction. This was adapted to the Kolb Cycle, where the experience of ‘feeling,’ established from the interactions with the patients, enhances the students’ learning to eventually allow them to reflect on their learning guided by their respective preceptors, as shown in this study. This permitted further thinking and evaluation of what they could have improved on for future and better implications [3,10]. However, confounding bias was apparent in this study as the development of greater rapport in an interview fostered a larger sense of meaning from those interactions. This would enhance the perceived effectiveness value, but whether the communication skills would improve more through different types of patients is uncertain.

**Figure 5:**
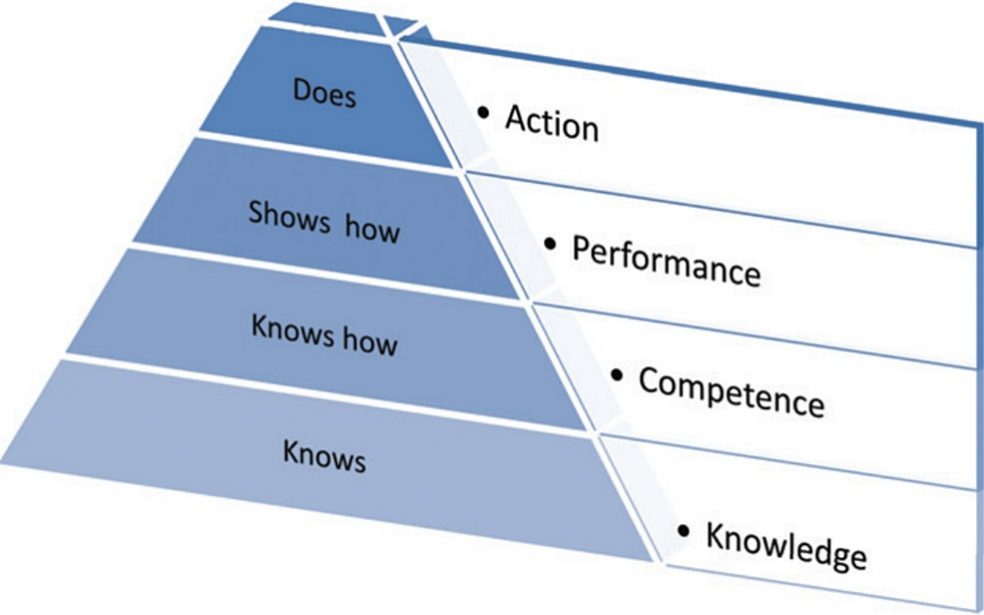
Miller’s Pyramid after Miller 1990 Ref no- [10] Permission to use image granted.

Jabeen [5] study was done in an assessment method whereby their performance was analyzed, and a Mini-CEX Score measured their ‘effectiveness’ of communication skills training [5]. This could be associated with Miller’s pyramid after Miller 1990 (Figure 5), where the outcome of the training is intended to take place in the workplace [10]. All the students have the base knowledge and competency. Researchers can predict the future actions of the medical students who will one day work as qualified doctors in their workplace based on their clinical performance while still in medical school. However, a confounding bias can be analyzed as the simulated patients were the nursing students trained instead of healthy volunteers from the general population [5]. Students’ performance might be falsely enhanced because the simulated patients are very well-trained with extensive medical knowledge, which might hinder students from learning from mistakes.

**Figure 6:**
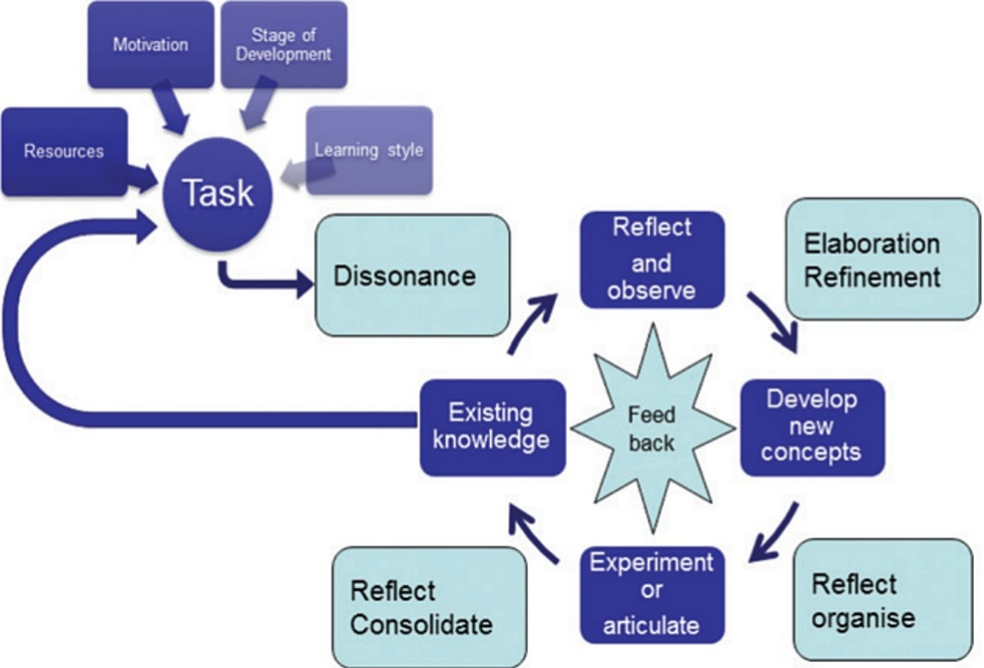
A Proposed Model of Adult Learning Ref no- [10] Permission to use image granted.

In a study done by Elley et al. [4], the type of adult learning theory (Figure 6) that was adopted was the proposed model of adult learning [10]. This was evident whereby the students had a dissonance phase as the knowledge was challenged in the patients’ interaction. When engaging with the patients, students would have gone through their refinement phase to determine possible explanations of the presenting complaint. They would restructure their ideas in the organization phase to understand which differential diagnosis the patient would most and least likely have based on the entire history. After the interview, the students were given valuable feedback by the student observer and actor. Finally, in the consolidation phase, the students would reflect on the feedback given to improve their communication skills for future interactions [4,10].

**Figure 7:**
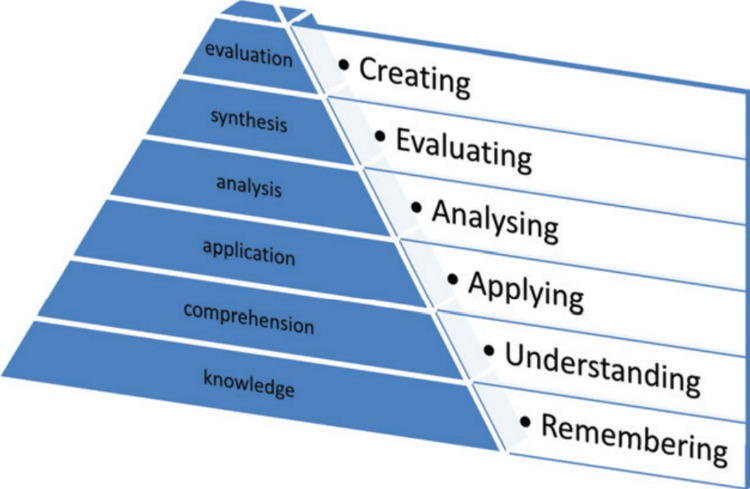
Bloom’s Taxonomy Ref no- [10] Permission to use image granted.

The Bokken et al. [2] study was done by interviewing some students on their thoughts and ideas about the effectiveness of communications skills training using simulated patients compared to real patients since they had prior exposure to both types of patients. This method would coincide with Bloom’s taxonomy learning theory (Figure7) [2,10]. The medical students had the knowledge and comprehension of their course, and they have applied it in the hospital settings in their clinical years with real patients. Additionally, they would have been exposed to SPs in the clinical skills center during their pre-clinical years [2]. They then analyzed the difference in both types of patients and evaluated which method would work best in the workplace. Hence, they created their style of approach to patients’ interactions, including physical examinations.

Application of results: In these studies, participants were recruited from the United States of America, Pakistan, New Zealand, and the Netherlands [2-5]. A non-probability sample was used by Jabeen [5] whereby all the fourth-year students in Shifa College of Medicine, Islamabad, were used without stating any variation of gender or demography. At the same time, Bokken et al. [2] study was conducted in a specific set of Maastricht Medical School, which differs from many medical schools in the United States (U.S.) with regards to the use of patient types. In other words, many U.S. medical schools introduced SPs and VOs simultaneously in the first year of the curriculum [2]. Hence, due to this difference, some results could not be generalized to the U.S. settings and other populations. Even though Clever et al. [3] study was conducted in the U.S., the subjects were in their first year and lacked medical knowledge when taking histories. This would not be a fair comparison. Therefore, the outcomes from the former would be easily applied to the local population. Furthermore, due to a short clinical attachment, it would be hard to establish the difference in patients’ outcomes and the long-term effects of different modes of teaching. However, clinical knowledge of diagnostic tools and management skills could be determined [4].

## Conclusions

It is apparent that SPs are more useful for pre-clinical years, intimate examination, and giving instructions regarding physical examination as it provides students a safe environment to make mistakes and learn from them. On the other hand, VOs are put to better use in clinical years to incorporate more medical aspects such as obtaining differential diagnosis and management of illness with additional training in procedural techniques and preparing students to be safe doctors in the future.

In summary, the objectives of this review were met as the measurement of ‘effectiveness’ of communication skills training is compared and established based on several adult learning theories using different types of patients. Its effectiveness is evident as both SPs and VOs play a vital role in medical students’ communication skills. This could be used to redefine the training scheme for medical students by introducing different types of patients based on their study progression and topic of discussion.
